# Rhesus Macaque Theta Defensins Suppress Inflammatory Cytokines and Enhance Survival in Mouse Models of Bacteremic Sepsis

**DOI:** 10.1371/journal.pone.0051337

**Published:** 2012-12-06

**Authors:** Justin B. Schaal, Dat Tran, Patti Tran, George Ösapay, Katie Trinh, Kevin D. Roberts, Kathleen M. Brasky, Prasad Tongaonkar, André J. Ouellette, Michael E. Selsted

**Affiliations:** 1 Department of Pathology and Laboratory Medicine, Keck School of Medicine, University of Southern California, Los Angeles, California, United States of America; 2 Kenneth Norris Comprehensive Cancer Center, University of Southern California, Los Angeles, California, United States of America; 3 Department of Pathology and Laboratory Medicine, University of California Irvine, Irvine, California, United States of America; 4 Texas Biomedical Research Institute, San Antonio, Texas, United States of America; Centre de Recherche Public de la Santé (CRP-Santé), Luxembourg

## Abstract

Theta-defensins (θ-defensins) are macrocyclic antimicrobial peptides expressed in leukocytes of Old World monkeys. The peptides are broad spectrum microbicides *in vitro* and numerous θ-defensin isoforms have been identified in granulocytes of rhesus macaques and Olive baboons. Several mammalian α- and β-defensins, genetically related to θ-defensins, have proinflammatory and immune-activating properties that bridge innate and acquired immunity. In the current study we analyzed the immunoregulatory properties of rhesus θ-defensins 1–5 (RTDs 1–5). RTD-1, the most abundant θ-defensin in macaques, reduced the levels of TNF, IL-1α, IL-1β, IL-6, and IL-8 secreted by blood leukocytes stimulated by several TLR agonists. RTDs 1–5 suppressed levels of soluble TNF released by bacteria- or LPS-stimulated blood leukocytes and THP-1 monocytes. Despite their highly conserved conformation and amino acid sequences, the anti-TNF activities of RTDs 1–5 varied by as much as 10-fold. Systemically administered RTD-1 was non-toxic for BALB/c mice, and escalating intravenous doses were well tolerated and non-immunogenic in adult chimpanzees. The peptide was highly stable in serum and plasma. Single dose administration of RTD-1 at 5 mg/kg significantly improved survival of BALB/c mice with *E. coli* peritonitis and cecal ligation-and-puncture induced polymicrobial sepsis. Peptide treatment reduced serum levels of several inflammatory cytokines/chemokines in bacteremic animals. Collectively, these results indicate that the anti-inflammatory properties of θ-defensins *in vitro* and *in vivo* are mediated by the suppression of numerous proinflammatory cytokines and blockade of TNF release may be a primary effect.

## Introduction

Antimicrobial peptides play a major role in host defense functions of mammalian granulocytes. Defensins, expressed in leukocytes and/or epithelia of most mammals studied, are 2–4.5 kDa cationic peptides that are further divided into three structural families (α-, β-, and θ-defensins) based on their distinctive tridisulfide motifs [1], [2]. α- and β-defensins, though genetically distinct, share similar peptide folds and are widely expressed in mammals including humans [3], [4]. Defensins of all three structural families were first recognized for their antimicrobial properties *in vitro.* Collectively, the antimicrobial spectrum of defensins includes bacteria, fungi, protozoa, and viruses [5], [6], [7]. Defensins also function as “alarmins” which elicit adaptive responses to infection and tissue injury [8], [9], [10].

θ-defensins have only been isolated from Old World monkeys and are absent in higher primates including gorillas, chimpanzees, and humans [1], [2], [11]. The peptides have a macrocyclic backbone that is post-translationally generated by pair-wise excision and head-to-tail splicing of two nine-residue segments derived from truncated α-defensin-related precursors [12]. The nonapeptides may be identical (homodimeric splicing) or derived from different precursors (heterodimeric splicing) thus amplifying the diversity of θ-defensin gene encoded products. Six rhesus macaque θ-defensin isoforms (RTDs 1–6) are expressed in neutrophils where they are packaged in cytoplasmic granules. Specific neutralization of RTDs in lysates of macaque neutrophil granules markedly reduced the antimicrobial activities of this preparation against *E. coli*, *S. aureus*, and *C. albicans*, indicating a prominent role for these peptides as components of the PMN antimicrobial armamentarium [13].

Intranasal administration of RTD-1 protected BALB/c mice from lethal infection by a mouse adapted strain of SARS-coronavirus (SARS-CoV) [14], despite the fact that the peptide did not neutralize the virus *in vitro*. The protective effect *in vivo* correlated with the reduction of pulmonary inflammation and the suppression of several pro-inflammatory cytokines in lung homogenates. To further characterize the immunoregulatory properties of θ-defensins, we analyzed the effects of natural θ-defensin isoforms on cytokine/chemokine responses in stimulated human leukocytes and THP-1 monocytes, and tested the efficacy of RTD-1 in two mouse models of bacteremic sepsis. The results of these studies also demonstrate that cyclic θ-defensins have sequence-specific anti-inflammatory properties that distinguish them from human neutrophil α-defensins.

## Materials and Methods

### Ethics Statement

#### Human subjects

Blood was obtained from healthy adult volunteers who provided written consent to participate. The study and consent form were approved by the Institutional Review Board at USC (protocol #HS-09-00280).

#### Animal studies

All animal studies were approved by the Institutional Animal Care and Use Committees where studies were performed: UC Irvine (protocol #2451; mouse studies), University of Southern California (protocol #11355; mouse studies), and Texas Biomedical Research Institute (protocol #1119PT0; chimpanzee study). Approved anesthetics were used for surgeries, recommended analgesics were used for post-operative care, and every effort was made to minimize suffering. Chimpanzees were housed in social groups in indoor/outdoor housing and cared for in accordance with the U.S. Public Health Service Guide for the Care and Use of Laboratory Animals and the U.S. Animal Welfare Act. They were fed standard monkey chow supplemented with fruits and vegetables twice daily. Potable water is available to each enclosure using lixit mechanisms. There is a very active environmental enrichment program for all animals and animal training is implemented to reduce stress and accomplish animal husbandry procedures cooperatively. For this study blood samples and compound administration were performed in anesthetized animals so any suffering was mitigated. No primates were sacrificed in this study. All animals were returned to their respective groups upon recovery from anesthesia.

### Peptide Reagents

The hydrochloride salts of RTDs 1–5 (>98%) were synthesized as described previously [12], [15], [16]. Human neutrophil α-defensins (physiologic mixture or HNP 1–3, or purified HNP-2) were purified from human neutrophils as previously described [17]. Stock solutions of each peptide (0.5–1.0 mg/ml) were prepared in 0.01% acetic acid (HOAc) for *in vitro* analyses or 0.85 M NaCl for administration to animals.

### Bacteria and TLR Agonists

*Staphylococcus aureus* 502a and *Escherichia coli* K12 were obtained from ATCC (Manassas, VA). A clinical isolate of *E. coli* was obtained from the clinical laboratory of the University of California Irvine Medical Center. Bacteria were cultured from single colonies and harvested as previously described [18]. Bacteria were washed and suspended in either 10 mM piperazine-N,N′-bis(2-ethane-sulfonic acid) (PIPES), pH 7.4 or phosphate buffered saline (PBS). Bacterial density was determined by spectrophotometric absorbance at 620 nm and correlated with colony forming units on tryptic soy agar plates. Toll-like receptor (TLR) agonists were obtained from Invivogen (San Diego, CA) and used as recommended by the manufacturer.

### Cell Culture

THP-1 monocytic cells (ATCC, Manassas, VA) were grown and maintained in RPMI-1640 medium containing 10% fetal bovine serum and penicillin/streptomycin. Cells were harvested by centrifugation, washed with RPMI-1640 medium and suspended at 5×10^5^ cells/ml in fresh medium containing 5% human EDTA plasma.

### Cytokine/Chemokine Release Assays

EDTA-anticoagulated blood was obtained from healthy adult volunteers as approved by the Institutional Review Board at USC (protocol # HS-09-00280). Peripheral blood leukocytes (PBL) were harvested from 10–15 ml whole blood after centrifugation at 200× g. Cells were washed twice with 3–5 ml of RPMI, counted with a hemocytometer, and suspended to a cell density of 5×10^5^ cells/ml in RPMI +5% human EDTA-plasma. PBLs were stimulated for 4 h with either 0.9 µg/ml ssRNA40, 30 ng/ml *S. typhimurium* flagellin, 3.3×10^7^ CFU/ml heat-killed *L. monocytogenes* (HKLM), 3 ng/ml *E. coli* K12 LPS, or 100 CFU/ml of the clinical *E. coli* isolate. Cytokines/chemokines were quantified using a Milliplex MAP kit on a BioRad Bioplex HTF Luminex reader at the Beckman Center for Immune Monitoring at USC-Norris Cancer Center.

### TNF Assay

Peptides (RTDs 1–5, human neutrophil α-defensins) were aliquoted into wells of pyrogen-free 12- or 24-well plates wherein final peptide concentrations were 0–10 µg/ml. Wells were inoculated with 1×10^5^ CFU/ml *S. aureus* or 100 CFU/ml *E. coli,* or 1–3 ng/ml of *E. coli* K12 LPS. Samples (5–10 µl) containing peptide or bacteria/LPS were placed on opposite sides of the plate well. Mixing commenced with the addition of 0.5 to 2.0 ml of 1∶10 diluted blood, PBLs (5×10^5^/ml), or THP-1 cells (2–5×10^5^ cells/ml). Plates were incubated at 37°C in 5% CO_2_ for 4 h with gentle mixing. In other experiments, 1∶10 blood/RPMI, *E. coli* cells, and 10 µg/ml of RTD-1 were pre-incubated in binary combinations for up to 120 minutes, followed by further incubation for 4 h for TNF release. Incubation mixtures were centrifuged at 200× g for 10 min at 22°C and supernatants were clarified by centrifugation at 23,000× g for 15 min at 4°C. Supernatant TNF was quantified by sandwich ELISA (OptEIA II; BD Biosciences; Hu TNF-α, Invitrogen) per suppliers’ directions, using a SpectraMax Plate Reader. In control experiments, RTD-1 had no effect on TNF ELISA standard curves.

### LPS Neutralization Assay

*E. coli* 0111:B4 LPS (Lonza), dissolved in endotoxin-free water (2 effective units/ml) was incubated with 0–10 µg/ml of RTD-1 or 0–10 µg/ml polymyxin B (Sigma) dissolved in 0.01% HOAc in a 50 µl reaction mixture for 10 min at 37°C. To each sample 25 µl of Limulus amoebocyte lysate (LAL; Lonza) was added and incubated for 10 min at 37°C after which 100 µl of LAL substrate was added and incubated for 6 min at 37°C. Reactions were quenched with 50 µl of 25% HOAc and read spectrophotometrically at 405 nm.

### RTD-1 Stability Analysis

RTD-1 (50 µg/ml final concentration) was incubated at 37°C in freshly prepared human serum, EDTA plasma, or 50 mg/ml human serum albumin in PBS. Aliquots were removed at 24 hour intervals for up to 72 h and acidified by addition of HOAc (10% final concentration). Peptide was quantified by sequential solid phase extraction on Strata X resin and quantitative RP-HPLC as described previously [13].

### Animal Exposure Studies

Groups of four BALB/c mice received daily 0.5 ml subcutaneous injections containing 0, 2.5, 10, 40, or 160 mg of RTD-1 per kg body weight. Twenty four h after the fourth injection, mice were euthanized with CO_2_, blood was collected by cardiac puncture, and tissues were harvested and fixed in 10% buffered formalin. Histopathologic examination was performed on sections of heart, lung, liver, spleen, kidney, and injection site skin and subcutaneous tissue. A basic metabolic panel (UC Davis-William R. Pritchard Veterinary Medical Teaching Hospital) was performed on serum samples obtained by cardiac puncture at the time of euthanasia.

Primate studies were conducted using two adult chimpanzees (15 y.o. male, 13 y.o. female). Animals were infused with escalating doses of RTD-1 (0.02, 0.1, 0.3, 1.0, and 3.0 mg/kg on days 0, 3, 7, 10, and 14, respectively) dissolved in 5 ml of pyrogen-free sterile saline. Blood specimens were obtained from each animal prior to RTD-1 administration and 30 and 60 min after peptide infusion. A comprehensive metabolic panel and complete blood count with differential was performed on each specimen. Samples from each animal were also obtained on days 21, 28, and 60 and similarly analyzed. Serum samples obtained at days 21, 28, and 60 were also analyzed for anti-RTD-1 antibody by dot blot analysis as described [13] employing anti-tetanus toxoid immunoreactivity as positive control.

### *E. coli* Peritonitis Model

Six to 8 week old BALB/c mice (Jackson Labs) were housed individually and provided with standard chow and water *ad libitum*. Peritonitis was induced by a single intraperitoneal injection of 8×10^8^ CFU of log-phase *E. coli* K12 in 500 µl PBS [19]. Mice were treated immediately with a single subcutaneous injection of 5 mg/kg RTD-1 in 0.5 ml of normal saline, or normal saline alone (control). Sham challenge was carried out with intraperitoneal injections of saline. Animals were monitored for 28 days and/or euthanized if they became moribund (counted as non-survivor). For cytokine/chemokine analyses, groups of four mice from each treatment group were euthanized at 0, 0.5, 1, 2, 4, and 12 hours following intraperitoneal challenge. Blood was collected by cardiac puncture into EDTA-tubes and plasma was prepared by a two-step centrifugation (200× g for 10 min followed by 23,000× g for 15 min). Soluble cytokines/chemokines were quantified using a mouse-specific Milliplex cytokine/chemokine kit as described above.

### Cecal-ligation/Puncture (CLP) Induced Sepsis

Polymicrobial peritonitis was induced in 6–8 week old BALB/c mice by CLP as described [20], [21]. Briefly, laparotomy was performed on anesthetized animals and the cecum was ligated below the ileocecal valve. Both walls of the cecum were punctured twice with an 18-gauge needle, and the surgical wound closed with 3–0 nylon suture. All animals fully recovered within 60 min. Four hours following CLP surgery, each animal received 150 µl of normal saline (n = 10) or 150 µl of normal saline containing 5 mg/kg of RTD-1 (n = 11) by tail vein injection. A separate group of mice (n = 5) was treated with RTD-1 as above but the single administration was delayed until 24 h after CLP surgery. Animal health was evaluated daily for 28 days. As above, mice were euthanized when they became moribund.

### Statistical Analyses

All values are expressed as mean +/− S.E.M except in experiments where n = 2 (as indicated in figure legends) where data are expressed as standard deviation. Significance of peptide effects on cytokines was determined by Student’s t test. Daily survival/death values were determined by χ^2^ test.

## Results

### θ-defensin Modulation of TLR-induced Cytokines/Chemokines

**Figure 1:**
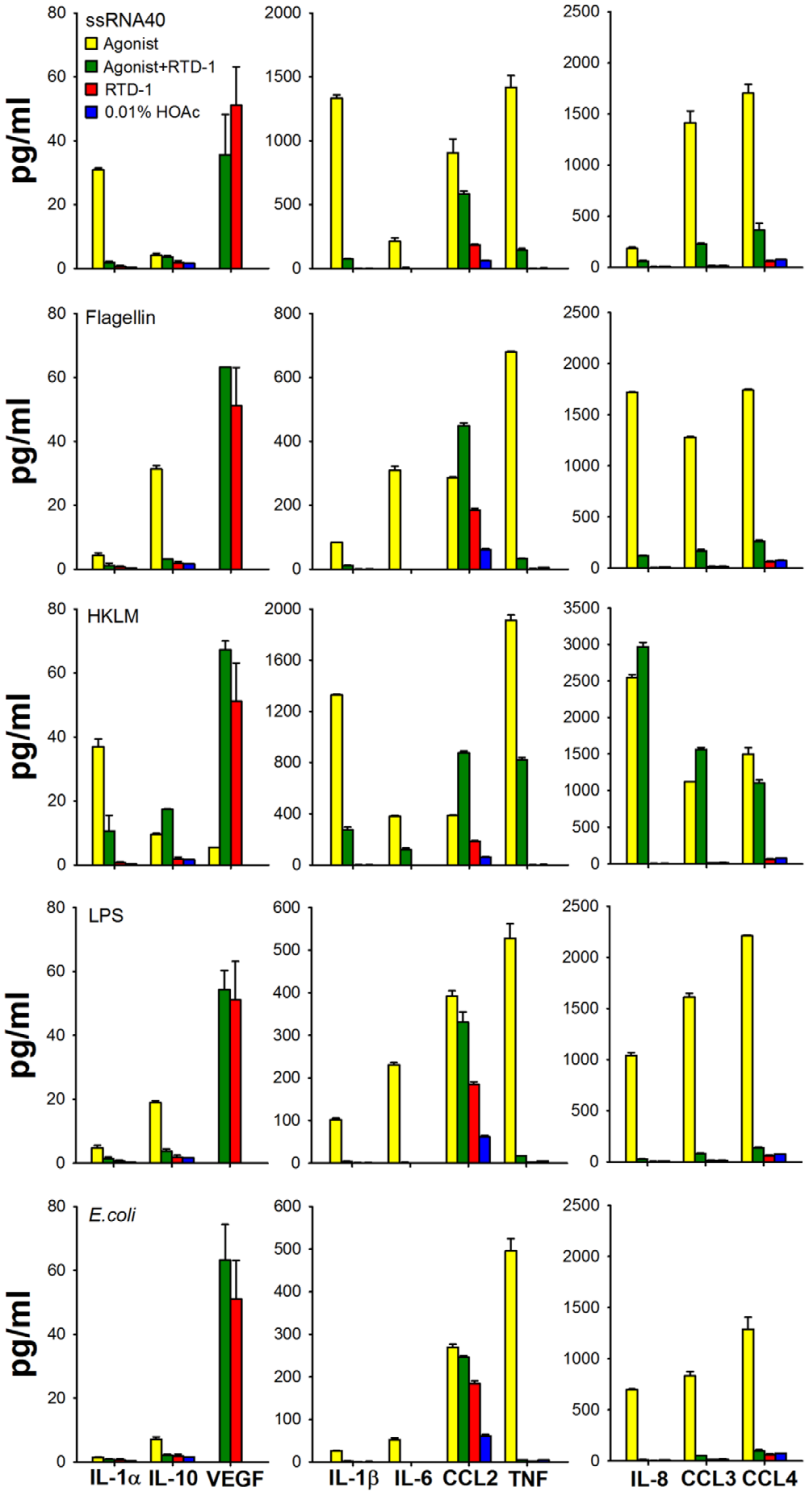
Effects of RTD-1 on stimulated release of cytokines/chemokines. Human buffy coat cells from EDTA-anti-coagulated blood were stimulated for 4 h with a panel of TLR agonists: 0.9 µg/ml ssRNA40, 30 ng/ml *S. typhimurium* flagellin, 3.3×10^7^ CFU/ml heat-killed *L. monocytogenes* (HKLM), 3 ng/ml *E. coli* K12 LPS, or 100 CFU/ml *E. coli* cells. Levels of ten soluble cytokines/chemokines were measured using a Milliplex MAP kit. Buffy coat cells stimulated with agonist (yellow), with +10 µg/ml RTD-1 (green), 10 µg/ml RTD-1 alone (red), and solvent control 0.01% HOAc (blue).

In a previous study, RTD-1 protected mice from lethal SARS-CoV via mechanisms that were independent of an antiviral effect, as RTD-1 was not virus neutralizing. Rather, RTD-1 administration appeared to protect infected animals by reducing pulmonary inflammation and suppressing IL-1α, IL-1β, IL-6, IL-12, CXCL1 (KC), CCL2 (MCP-1), CCL3 (MIP-1α), and CCL5 (RANTES) 2–4 days post-infection [14]. To determine how RTD-1 modulates inflammatory responses of human cells, peripheral blood leukocytes (PBL) were incubated with different TLR agonists with and without 10 µg/ml of RTD-1 and evaluated for the release of soluble cytokines/chemokines. We initially tested the effect of RTD-1 on ssRNA (TLR8 agonist) induced responses of PBLs to compare human cell responses with those obtained in mouse SARS-CoV (a single stranded RNA virus) pneumonitis. As shown in Figure 1, ssRNA stimulation of human leukocytes induced cytokines/chemokines similar to those observed in murine SARS-CoV pneumonitis including IL-1α, IL-1β, IL-6, TNF, CXCL8 (IL-8), CCL2 (MCP-1), CCL3 (MIP-1α), and CCL4 (MIP-1β). The simultaneous addition of 10 µg/ml of RTD-1 reduced supernatant levels of each of the above listed cytokine/chemokines with suppression ranging from 68% (IL-8) to 95% (IL-1β).

RTD-1 also down regulated leukocyte release of cytokines/chemokines induced by other TLR agonists: HKLM (TLR2), flagellin (TLR5), LPS (TLR4) and live *E. coli* cells (Fig. 1). RTD-1 markedly reduced IL-1α, IL-1β, IL-6, TNF, CXCL8 (IL-8), CCL3 (MIP1α), and CCL4 (MIP1β) levels induced by HKLM (TLR2) and LPS (TLR4) and by *E. coli* cells, similar to the effect observed with RTD-1 treatment of ssRNA-stimulated cells (Fig. 1). As expected, the effects of RTD-1 on leukocyte responses to LPS and *E. coli* were similar. The effect of RTD-1 on HKLM-stimulated cells differed somewhat from the peptide’s effect on leukocytes activated by other agonists. Although RTD-1 treatment of HKLM-stimulated cells markedly reduced TNF, IL-1α, IL-1β, and IL-6 levels, similar to the effects observed with other agonists, there was ∼ 40% increase in IL-10 and no suppression of induced levels of chemokines CXCL8 (IL-8), CCL2 (MCP-1), CCL3 (MIP-1α), or CCL4 (MIP-1β). RTD-1 alone had little effect on cytokine/chemokine release by leukocytes with the exception of VEGF and to a lesser degree CCL2 (MCP-1).

### RTD-1 Inhibits TNF Release in Human Blood

**Figure 2:**
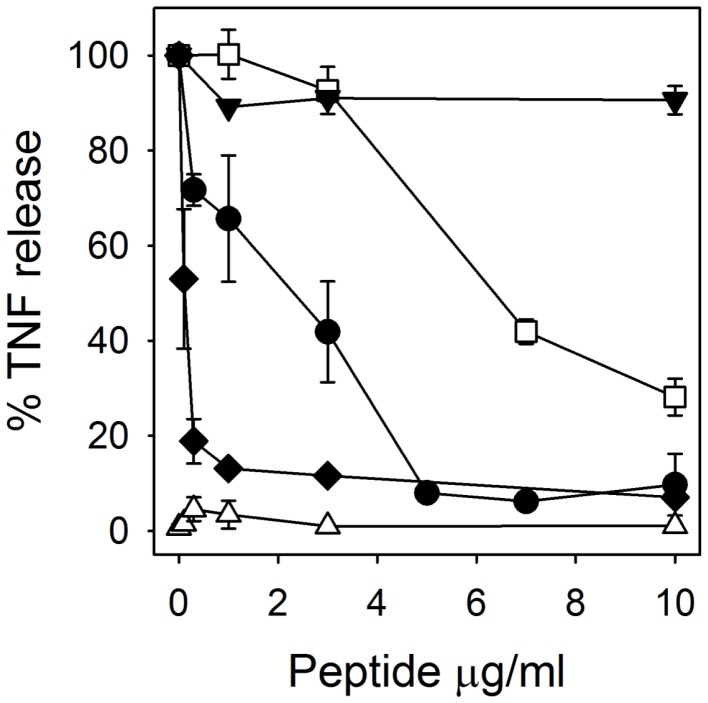
RTD-1 inhibits the release of soluble TNF in whole blood. Human EDTA-anti-coagulated blood diluted 1∶10 in RPMI, was stimulated for 4 h with live *E. coli* cells (•), *S. aureus* 502a (⧫), or *E. coli* K12 LPS (□) with simultaneous addition of RTD-1 at the concentrations indicated. *E. coli*-stimulated blood was similarly analyzed for effects of a natural mixture of HNP 1–3 (▾). Blood incubated for 4 h with RTD-1 alone shown as (▵). N = 2–7 for each experiment.

Of the cytokines evaluated in the experiments described above, TNF is regarded as the earliest inflammatory response signal and in this regard functions as a trigger of numerous downstream inflammatory responses [22], [23]. To investigate the effect of RTD-1 on TNF release stimulated by bacterial antigens, anticoagulated human blood was inoculated with *E. coli* or *S. aureus*, or stimulated with *E. coli* K12 LPS in the presence of varied concentrations of the peptide. In each of these mixtures, RTD-1 blocked stimulated TNF released in a dose-dependent manner, with ED50’s of ∼0.1 µg/ml for *S. aureus*, ∼2 µg/ml for *E. coli,* and ∼ 5 µg/ml for LPS (Fig. 2). RTD-1 alone had no effect on TNF release by blood leukocytes. Of note, a physiologic mixture of human α-defensins (HNP 1–3) had no TNF-suppressive effects on *E. coli*-stimulated PBLs (Fig. 2). In keeping with previous studies [18], no cytotoxicity was detected in these incubations as evidenced by a lack of trypan blue staining of leukocytes.

### RTD-1 Binding to LPS

**Figure 3:**
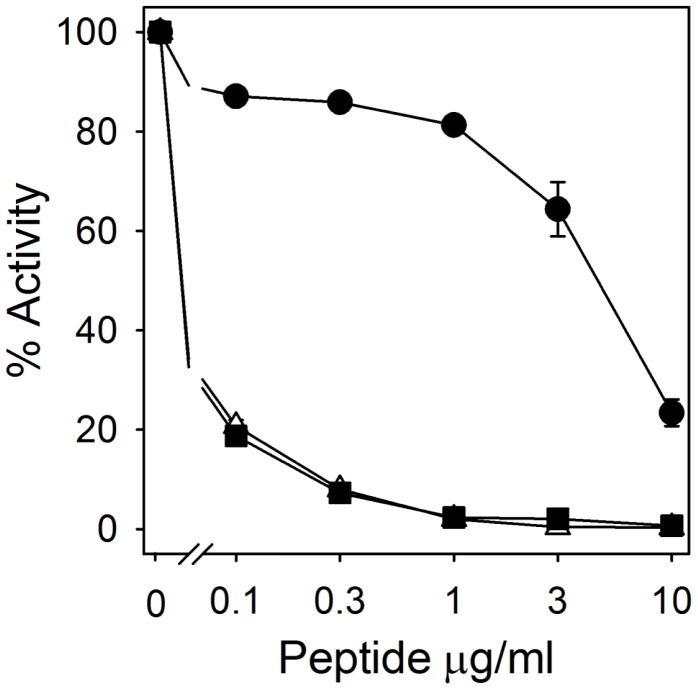
RTD-1 is ineffective in neutralizing LPS. The biological activity of LPS in the presence of 0–10 µg/ml of RTD-1 (•), polymyxin B (▪), or 1∶1 mixtures of RTD-1 and polymyxin B (▵) was determined by limulus amoebocyte lysate assay. Data are the average on N = 4 experiments.

The inhibition of LPS-induced cytokine/chemokine release by RTD-1 (Figs. 1 and 2) suggested that the peptide might bind this agonist, as has been demonstrated for other anti-microbial peptides [24], [25]. RTD-1 was ineffective in neutralizing *E. coli* LPS, being 50–100 fold less effective than polymyxin B (Fig. 3). Moreover, mixing of RTD-1 with polymyxin B showed no additive or antagonistic effects (Fig. 3). These results indicate that RTD-1 inhibits endotoxin-stimulated release of TNF via mechanisms other than LPS neutralization. This is consistent with the finding that RTD-1 was a potent inhibitor of TNF release by PBLs stimulated with multiple TLR agonists (Figs. 1 & 2).

### Temporal Analysis of RTD-1-Mediated Blockade of TNF Release

**Figure 4:**
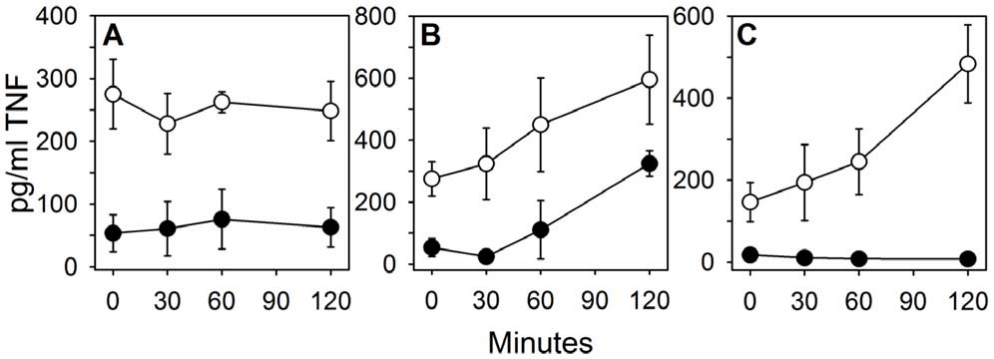
Temporal analysis of TNF release from *E. coli* stimulated blood. A : Blood diluted 1∶10 in RPMI was pre-incubated with 10 µg/ml of RTD-1 (•) or solvent (○) for 0, 30, 60, and 120 min after which 100 CFU/ml of live *E. coli* cells were added and incubated for another 4 h. **B**: Diluted blood in RPMI was pre-stimulated with 100 CFU/ml *E. coli* for 0, 30, 60, and 120 min followed by addition of 10 µg/ml of RTD-1 (•) or solvent (○) and incubated for an additional 4 h. **C**: 100 CFU/ml *E. coli* cells were pre-treated with 10 µg/ml of RTD-1 (•) or 0.01% HOAc (○) in RPMI for 0, 30, 60, and 120 min after which whole blood (1∶10 dilution final) was added and incubated for 4 h. After the secondary 4 h incubations in A–C, supernatant TNF levels were determined by ELISA. Data are average of N = 2–3 experiments.

In the experiments summarized in Figures 1 and 2, RTD-1 modulation of leukocyte responses was analyzed following simultaneous mixing of peptide, inflammatory stimulus, and leukocytes or whole blood. To better understand the sequence of peptide-mediated TNF blockade, mixing experiments were performed wherein blood, peptide, and *E. coli* cells were pre-incubated in varied combinations (see *Methods*), and TNF release was quantified as described above. Pre-incubation of RTD-1 with blood for up to 2 h had no effect on the magnitude of TNF-release inhibition which was quite stable (70–80%) over the pre-incubation time course (Fig. 4A). This is consistent with the effect of RTD-1 on TNF release when RTD-1, *E. coli*, and blood were mixed simultaneously (Fig. 2). In contrast, pre-mixing of blood and *E. coli* cells for up to 2 h, followed by addition of RTD-1, showed a time-dependent increase of TNF release in both control and peptide containing mixtures (Fig. 4B). However, at each time point the presence of RTD-1 reduced TNF levels (46–93%; Fig. 4B). These results indicate that RTD-1-mediated blockade of *E. coli*-stimulated TNF release is very rapid, occurring immediately after addition of the peptide to leukocyte-containing mixtures (Figs. 2 and 4). When *E. coli* cells were incubated with RTD-1 prior to addition of blood, complete blockade of TNF release was observed at all time-points (Fig. 4C), whereas *E. coli* alone elicited increasing levels of TNF as a function of pre-incubation time. During the 120 minute pre-incubation interval, bacterial counts increased 3.2-fold, consistent with the temporal rise in inducible TNF release following the addition of blood (Fig. 4C). On the other hand, the absence of viable bacteria in samples containing RTD-1 revealed that *E. coli* was efficiently killed by the peptide by within 30 min. The potent inhibition of *E. coli*-stimulated TNF release by blood leukocytes at T = 0 in this experiment further demonstrates the rapid blockade of bacteria-stimulated TNF release by RTD-1 (Fig. 4C). Moreover, the bactericidal effect of RTD-1 terminates further production of bacterial antigen which, based on bacterial cell numbers, tripled in 2 h in the absence of RTD-1 (Fig. 4C). Taken together, the data summarized in Figures 2 and 4 suggest that RTD-1 interacts with leukocytes to suppress TNF in response to microbial antigens.

### Anti-TNF Activities of θ-Defensin Isoforms

**Figure 5:**
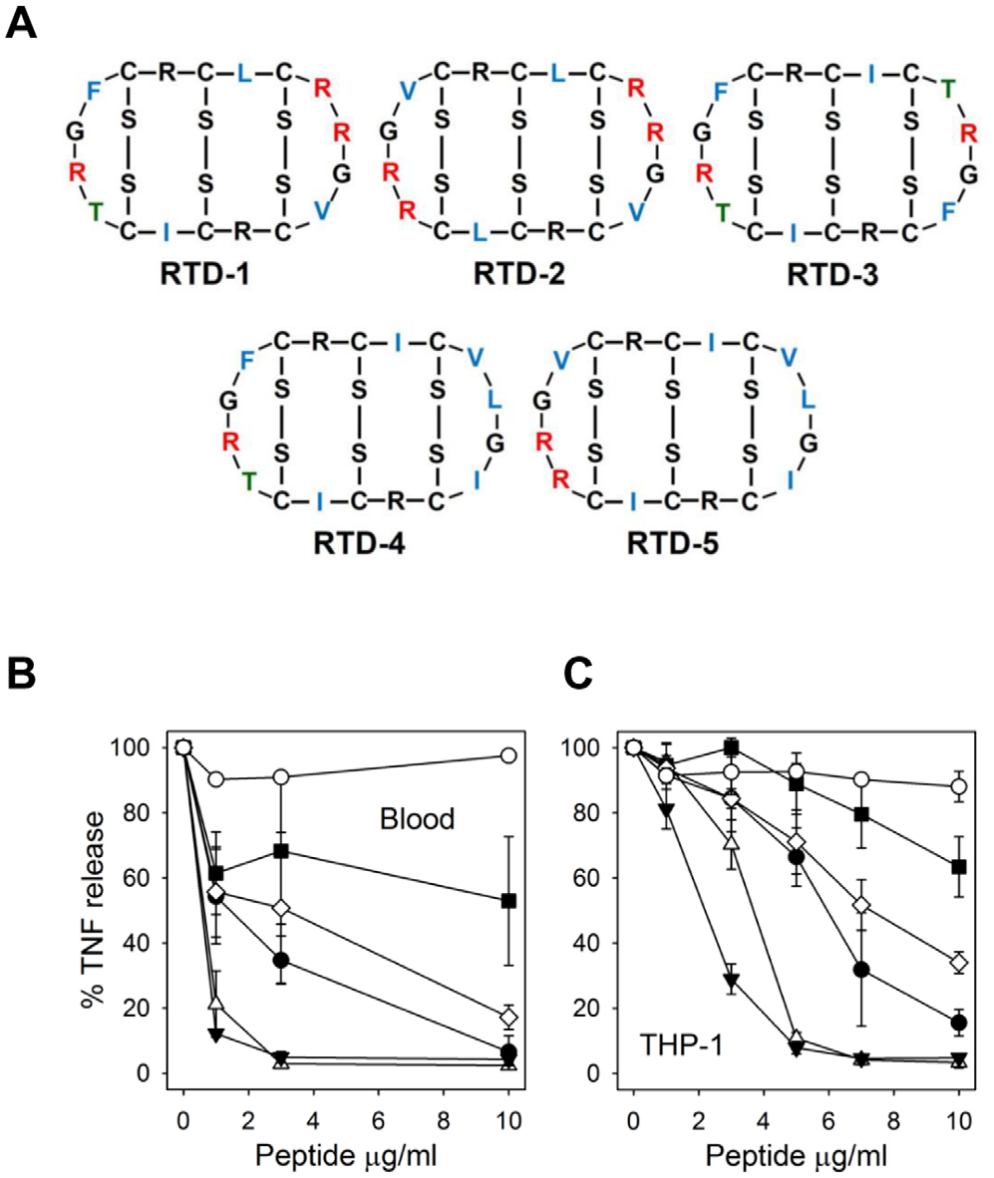
RTD isoforms differentially inhibit TNF release by *E. coli*- and LPS-stimulated human blood and THP-1 monocytes. A. Covalent structures of RTD 1–5 are shown with invariant residues in black, variable Arg (red), variable hydrophobic residues (green), and variable Thr (blue). B. Human EDTA-anticoagulated blood diluted 1∶10 in RPMI was stimulated for 4 h with live *E. coli* (100 CFU/ml) and RTD-1 (•), RTD-2(▵), RTD-3 (▪), RTD-4 (⋄), RTD-5 (▾), or HNP-2 (○) at the indicated concentrations. C. THP-1 cells in RPMI +5% human EDTA-plasma were stimulated with 1 ng/ml K12 LPS and incubated with RTDs 1–5 and HNP-2 as described in (B). For both (B) and (C), supernatant TNF levels were determined by ELISA. N = 2 experiments.

RTD-1 is the most abundant of six θ-defensin isoforms expressed in neutrophils and monocytes of rhesus monkeys [13]. To determine the relative anti-inflammatory activities of other θ-defensins isoforms (RTDs 2–5; Fig. 5A), we analyzed the effects of these peptides on TNF levels in *E. coli*-blood assays described above. As shown in Figure 5B, all θ-defensin isoforms suppressed supernatant TNF levels with potencies, based on estimations of IC50, ranging from 1–10 µg/ml (0.5–5 µM). RTDs 2 and 5 were substantially more effective than RTD-1, whereas RTDs 3 and 4 were less active. Analogous experiments were performed to assess the effects of θ-defensin isoforms on LPS-stimulated THP-1 monocytes. In these experiments RTD 1–5 inhibited stimulated TNF release and the IC50s once again varied ca. 10 fold (Fig. 5C). Of note, the hierarchy of anti-TNF potencies was the same as that obtained in *E. coli*-stimulated blood experiments, i.e., RTD-5>2>1>4>3. Human α-defensin HNP-2 had no inhibitory effect on TNF release in either assay (Fig. 5B & C; also see Fig. 2), and a physiologic mixture of HNP 1–3 also lacked TNF inhibitory activity (data not shown). In each of the *in vitro* experiments described above, we confirmed that the reduction of cytokine expression was not the result of cytotoxic effects on the target cells as trypan blue staining confirmed that cell viability was >99% at the end of each incubation interval. Lack of cytotoxicity was further confirmed by the finding that VEGF-A expression increased following RTD-1 treatment (Fig. 1; discussed further below).

### RTD-1 is Non-toxic and Non-immunogenic

**Figure 6:**
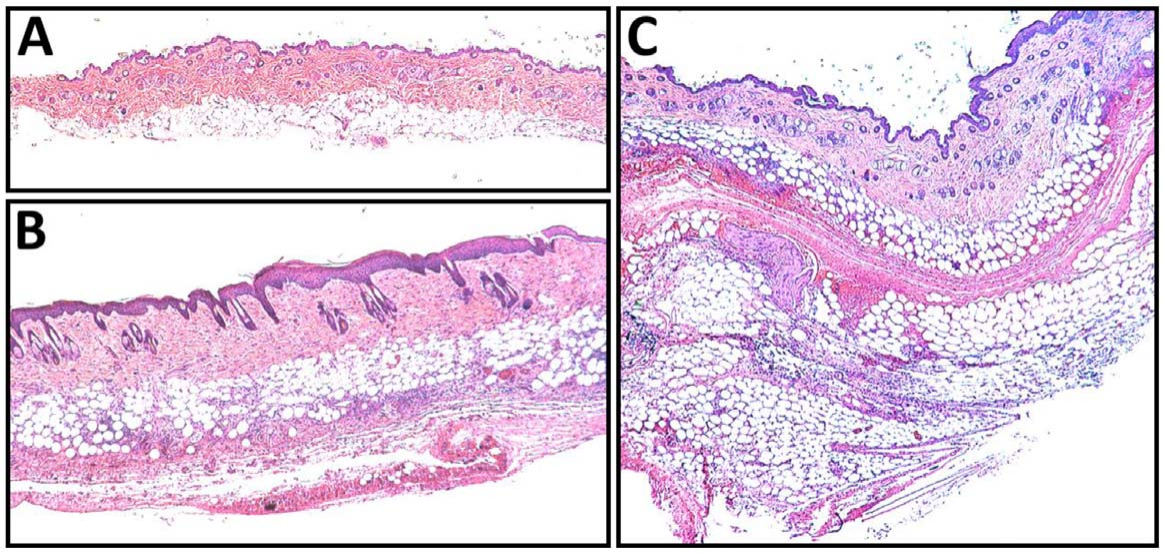
High-dose RTD-1 injection site reaction in Balb/c mice. Hematoxylin-eosin stained sections of normal (A) and indurated (B, C) skin demonstrate that multiple injections with 40 mg/kg RTD-1 (B) or 160 mg/kg RTD-1 (C) produce a lobular panniculitis with fat necrosis. These changes were absent in animals receiving multiple injections of 0, 2.5, or 10 mg/kg of RTD-1.

In earlier studies RTD-1 was non-toxic to host cells *in vitro* [18] and was well tolerated when administered intranasally to mice [14]. In the current study, no acute toxicity was observed in animals receiving subcutaneous doses of RTD-1 (up to 160 mg/kg, the highest dose tested), and serum chemistries in RTD-1-treated mice were indistinguishable from saline-treated controls. Histopathologic examination of lungs, kidneys, heart, liver, and spleen from RTD-1 treated animals showed no abnormalities after the 4 day dosing regimen at all peptide levels tested. The only detected tissue effect associated with RTD-1 administration was focal, non-erythematous swelling that appeared at the injection site (dorsal midline thoracic skin) in 4/4 mice (by day 3) in the 160 mg/kg group and 2/4 animals (at day 4) in the 40 mg/kg group. Histologic examination of affected tissue revealed lobular panniculitis with fat necrosis around the injection site in affected animals (Fig. 6).

Escalating doses of RTD-1 (0.2 to 3.0 mg/kg over 14 days) were administered intravenously to two adult chimpanzees and the animals were evaluated clinically and for effects on serum chemistries and hematologic parameters. No clinical or injection site effects were observed at any dosing level over the period of the study. Comprehensive metabolic panels and complete blood counts with differential revealed no abnormalities associated with peptide administration at any time point during the study, including those obtained after dosing was halted (day 14), i.e., at days 21, 28, and 60. Serum samples at each time point were also evaluated for anti-RTD-1 antibody by dot blot immunoassay [13] and for anti-tetanus toxoid antibody as positive control. No anti-RTD-1 antibody was detected in samples from either animal. In additional experiments, repetitive (8–15 injections) subcutaneous challenge of DA rats with RTD-1 produced no anti-RTD-1 antibody after 8 weeks (Schaal et. al, manuscript in preparation).

### RTD-1 Stability

The macrocyclic conformation of RTD-1 confers remarkable resistance to enzymatic degradation, a property that confounded initial attempts to determine the peptide’s covalent structure [12]. RTD-1 is completely stable to heating (100°C, 30 min) and extended storage at pH 2.0 (D. Tran & M.E. Selsted, unpublished data). We further evaluated RTD-1 for stability in human serum, EDTA-anticoagulated plasma, and 50 mg/ml human serum albumin, incubating the mixtures at 37°C for 72 h. Time zero concentrations of RTD-1, determined by quantitative RP-HPLC, of each mixture were identical within the limits of method precision. After 72 h of incubation, RTD-1 levels, relative to time zero, were 112% (serum), 93% (plasma), and 81% (albumin), demonstrating that the peptide is stable in biological fluids. Consistent with these data, RTD-1 is also highly stable in whole blood from humans, rats, and mice for at least 24 h (data not shown).

### Efficacy of RTD-1 in Mouse Peritonitis

**Figure 7:**
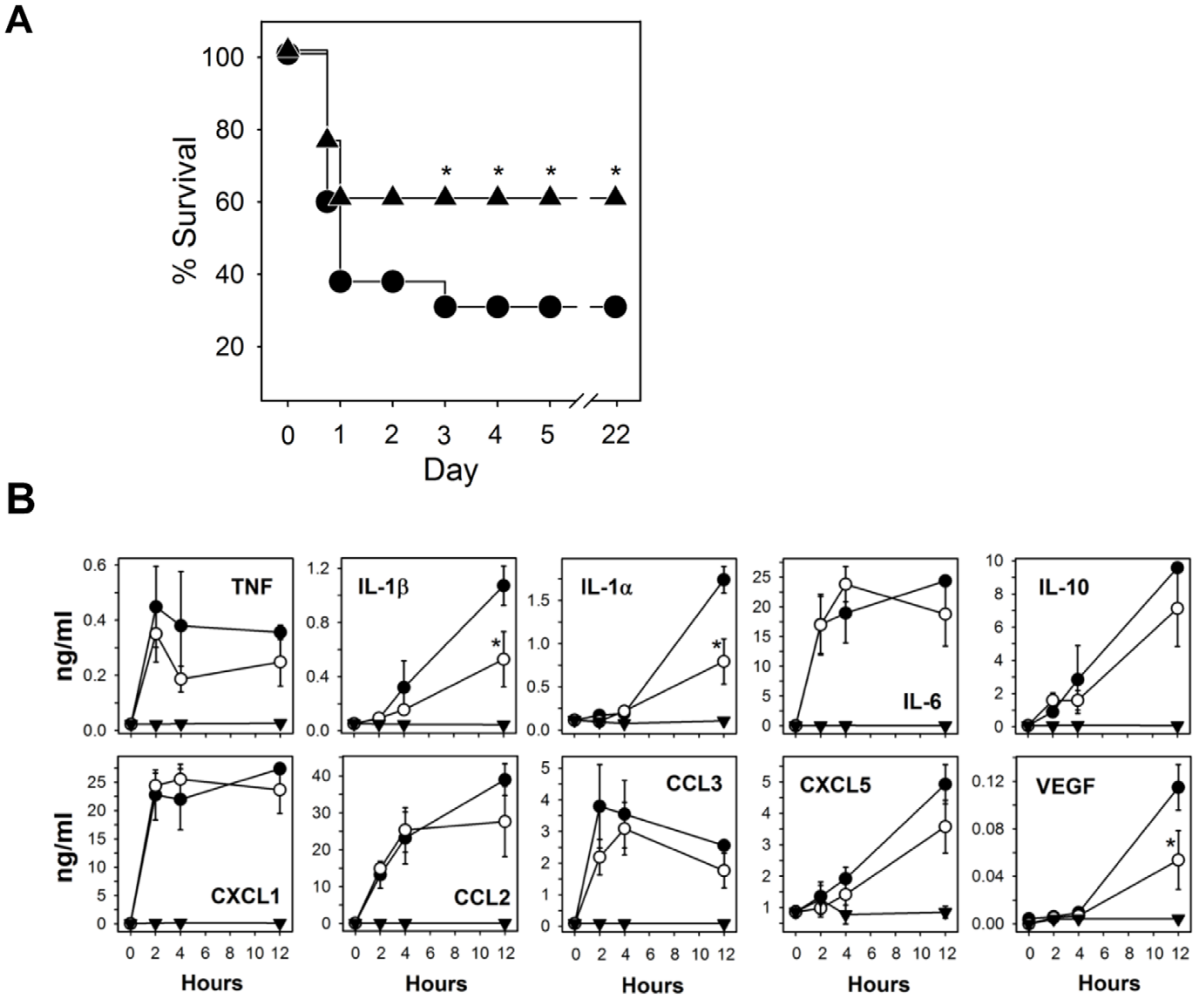
RTD-1 increases survival in *E. coli* peritonitis and modulates cytokine/chemokines. **A**. BALB/c mice were challenged with 8×10^8^ CFU *E. coli* K12 and treated simultaneously with s.c. injection of saline (•; n = 13) or 5 mg/kg RTD-1 (▴; n = 13). Endpoint survival data are plotted and were subjected to χ^2^ analysis and *P*-value was ≤0.017 *) by day 3 or later. **B**. Plasma cytokines/chemokines were quantified in blood obtained from animals euthanized (n = 4 for each time point) at 0, 2, 4, and 12 h after i.p. challenge and treatment with saline (•) or 5 mg/kg RTD-1 (○). Sham controls were injected with RTD-1 alone (▾). Cytokines/chemokines were quantified as described in *Methods* and results subjected to Student’s *t*-test; *P*≤0.05 (*).

We evaluated the effects of RTD-1 *in vivo* using two mouse models of bacterial peritonitis. A single subcutaneous dose of RTD-1 (5 mg/kg) significantly improved survival of mice infected intraperitoneally with live *E. coli* (Fig. 7). Animals in the peptide and saline controls that survived beyond day 3 were clinically normal and no further deaths occurred over the course of the trial (day 22; Fig. 7A). Plasma cytokine/chemokine levels in RTD-1 treated and untreated bacteremic mice were analyzed in cohorts of mice from each treatment group euthanized at 2, 4, and 12 hours post challenge/treatment. Untreated bacteremic animals had marked elevations in IL-1α, IL-1β, IL-6, IL-10, CXCL1, CCL2, CCL3, CXCL5, TNF, and VEGF (Fig. 7B). Treatment with RTD-1 resulted in reductions of all cytokines, but only the decreases of IL-1α, IL-1β, and VEGF were statistically significant (P<0.05). Cytokine/chemokines levels in uninfected, RTD-1 treated animals were unaltered compared to saline treated controls.

**Figure 8:**
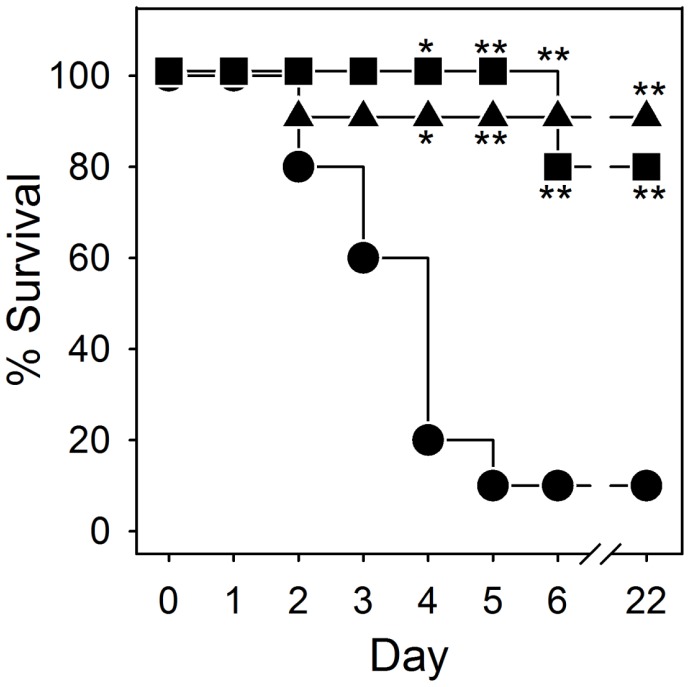
RTD-1 increases survival in a mouse model of polymicrobial sepsis. CLP was performed as described in *Methods* and animals were treated with i.v saline 4 h post CLP surgery, (•, n = 10), 5 mg/kg RTD-1 4 h post-CLP surgery (▴, n = 11), or RTD-1 24 h after CLP surgery (▪, n = 5). Endpoint survival data are plotted and were subjected to χ^2^ analysis and statistical significance are indicated with * for *P*<0.05 and ** for *P*<0.001.

We also evaluated the effects of RTD-1 in BALB/c mice rendered septic by cecal ligation and puncture [21]. While 90% of the saline treated animals died within 5 days of CLP surgery, a single i.v. dose of RTD-1 (5 mg/ml) administered 4 h after CLP surgery resulted in long term survival of 10 of 11 mice (Fig. 8). Surprisingly, 4 of 5 mice that were not treated until 24 h post CLP, also recovered and were clinically normal through the end of the trial.

## Discussion

The results of studies presented here reveal that θ-defensins possess potent anti-inflammatory properties *in vitro* and *in vivo*. In a previous study, RTD-1 suppressed pulmonary bronchiolitis and the levels of pro-inflammatory cytokines induced by SARS-CoV, an ssRNA virus. RTD-1 treatment of ssRNA-stimulated PBLs suppressed the levels of inflammatory cytokines and chemokines (Fig. 1), and the anti-inflammatory profile was similar to that obtained in the SARS-CoV pneumonitis model. RTD-1 also suppressed cytokine/chemokine release by PBLs stimulated with other TLR agonists, including those for TLRs 2, 4, and 5, and the peptide suppressed TNF release stimulated by both Gram-positive (*S. aureus*) and Gram-negative (*E. coli*) bacteria. Based on these findings we speculated that the effects of RTD-1 were due to the modulation of early interactions of leukocytes with inflammatory stimuli. RTD-1 was ineffective in neutralizing LPS, indicating that peptide binding of this TLR4 agonist is unlikely to be an important anti-inflammatory mechanism. This differentiates θ-defensins from other antimicrobial peptides that bind and neutralize LPS [24]–[26].

Pre-incubation experiments described above (Fig. 4) revealed that RTD-1 very rapidly blocked *E. coli*-stimulated TNF release in human blood, and temporal analyses of these mixing experiments implicated peptide-leukocyte interactions to be the critical determinant of TNF blockade. The time scale of the inhibitory effects (minutes) suggests that θ-defensin may disrupt the mobilization of TNF from the surface of stimulated cells. Since TNF plays a central role in triggering and sustaining inflammatory cascades [22], [23], [27], θ-defensin blockade of TNF may suppress subsequent inflammatory responses, thereby reducing levels of other inflammatory cytokines/chemokines *in vitro* (Fig. 1) and *in vivo* (Fig. 7B). Alternatively, θ-defensins may interrupt TNF autocrine circuits that amplify the effect of this acute phase cytokine. While suppression of RTD-mediated TNF release was a common feature of antigen-stimulated leukocyte responses *in vitro*, we have yet to identify downstream mechanisms that result in the down regulation of secondary pro-inflammatory cytokines/chemokines. In this context, the effects of inflammatory mediator blockade is highly complex and context dependent, and likely involves crosstalk of signaling factors that are differentially induced by distinct TLR agonists and other stimuli to produce protective and/or pathologic responses [28]–[30]. Thus it is not surprising that the immunomodulatory effects of RTD-1 varied as a function of inflammatory stimuli.

Correlation of *in vitro* effects, such as those analyzed using whole blood, PBLs, and monocyte-macrophages, with effects observed *in vivo* (e.g., sepsis models) must be interpreted with caution. In this regard, the relatively modest effects of systemically-administered RTD-1 on plasma cytokines in bacteremic mice contrasts with the dramatic down regulation of cytokines that occurred when leukocytes were treated with θ-defensins *in vitro;* this apparent discrepancy is observed in many if not most *in vitro*/*in vivo* model comparisons. It is evident that the pathways induced by θ-defensins *in vivo* (e.g., Fig. 7A and 8) require further investigation to delineate the mechanisms that confer efficacy in these models. In this context, RTD-1 treatment of PBLs produced a reproducible elevation of VEGF-A. However, the peptide had no such effect *in vivo*, and in fact significantly reduced VEGF-A levels in bacteremic mice (Fig. 7B). In the lung, both protective and pathologic roles have been ascribed to VEGF and its physiologic regulation appears to play a critical role in the outcome of pulmonary acute lung injury and acute respiratory distress syndrome [31]. The induction of leukocyte VEGF-A by RTD-1 may represent a new mechanism whereby circulating cells are stimulated to release this vascular growth factor by locally expressed θ-defensin. It is of interest to note the human neutrophil α-defensins inhibits VEGF-dependent neovascularization [32], [33], potentially highlighting another difference between α- and θ-defensins. Studies are underway to analyze the mechanisms underlying the induction of VEGF-A in θ-defensin stimulated leukocytes.

Despite the fact that all known θ-defensins have an invariant 10-amino acid core structure (Fig. 5A), five θ-defensin isoforms varied significantly in their blockade of *E. coli* or LPS-stimulated inflammatory responses, and the hierarchy of anti-TNF potencies was the same in these two cellular assays. There was no correlation between peptide charge and anti-TNF efficacy; in fact the most effective (RTD-5) and least effective (RTD-3) peptides both have a net charge of +4. Human α-defensins shared none of the anti-inflammatory properties observed with θ-defensins. This is not surprising, given the lack of structural similarity between α- and θ-defensins [2], and the fact that α-defensins possess pro-inflammatory properties that include up regulation of TNF and IL-1β expression by monocytes [34], stimulation of TNF, IL-6, and IL-12 expression in myeloid dendritic cells [35], and induction of IL-8 release by lung epithelial cells [36]–[38].

The macrocyclic structure of θ-defensins confers remarkable stability. RTD-1 was unmodified by incubation for up to 3 days in freshly prepared plasma or serum. RTD-1 was also well-tolerated when administered intravenously or subcutaneously to mice, rats, and chimpanzees. Following repeated injections, neither of two chimpanzees produced anti-RTD-1 antibody. The biocompatibility of RTD-1 enabled an evaluation of its therapeutic potential in animal models of systemic inflammation. The discovery of antimicrobial peptides and their proven roles in host defense has prompted studies to evaluate diverse peptides derived from human cells (LL-37 [39], [40]), ungulates (indolicidin [41])*** and sheep myeloid antimicrobial peptide (SMAP)-29 [42]), and pigs (protegrins [24], [43]) in preclinical bacteremia models. Protection against lethal bacteremia by LL-37 [40], indolicidin [44], and SMAP-29 [42] was observed following a single systemic administration of peptide at the time of bacterial challenge and therapeutic effects in each case were attributed to anti-endotoxic activities of the respective peptides. In contrast, in CLP sepsis, multiple doses of porcine protegrin PG-1 had little endotoxin-neutralizing effect and the treatment regimen produced no therapeutic effect compared to vehicle control [45]. Data presented here suggest that RTD-1 alters the course of disease in two models of bacteremic sepsis in a manner different from the above examples. Single dose administration of RTD-1 in either *E. coli* peritonitis or CLP sepsis produced a therapeutic response. In the former model, simultaneous but modest reductions in inflammatory cytokines were observed in RTD-1 treated animals. Surprisingly, a single dose of RTD-1 rescued mice rendered septic by CLP surgery even when treatment was delayed for 24 after peritonitis was induced. Efficacy of θ-defensins in these models appears to be independent of direct anti-endotoxic effects since the peptide was ineffective in blocking the effects of endotoxin in the limulus amoebocyte assay. Current studies are underway to further characterize the mechanistic bases for the immunomodulatory activities of θ-defensins *in vitro* and *in vivo*. The lack of immunogenicity and toxicity across species raises the possibility that θ-defensin may have utility as human therapeutics.
